# Supplementary material for: Genomic landscape of lymphatic malformations: a case series and response to the PI3Kα inhibitor alpelisib in an N-of-1 clinical trial
Source: eLife. 2022 Jul 5;11:e74510. doi: 10.7554/eLife.74510 (PMC9255965; doi:10.7554/eLife.74510)
Supplement: Supplementary file 1. — The genetic coding variants that exist in lymphatic malformation (LM) but do not exist in germline DNA. These pass MuTect2 quality filters (designed to call somatic variants only) and have three or more alternate reads. VAF, variant allele frequency; COSMIC, Catalogue Of Somatic Mutations In Cancer. [file elife-74510-supp1.docx]

**Supplementary Table 1. Somatic coding mutations identified from whole genome sequencing**

| **Gene_name** | **Protein_change** | **VAF** | **Alt_depth** | **Ref_depth** | **COSMIC** |
| --- | --- | --- | --- | --- | --- |
| HRNR 0.105 | p.T616A | 0.105 | 4 | 51 |  |
| OR2T3 | p.S247F | 0.171 | 8 | 53 |  |
| PLXDC2 | p.V396I | 0.167 | 4 | 22 |  |
| FOLH1 | p.R190W | 0.333 | 8 | 20 |  |
| IPO8 | p.M488fs | 0.125 | 3 | 28 |  |
| KRR1 | p.R134Q | 0.188 | 3 | 17 |  |
| KRTAP4-11 | p.L161V | 0.116 | 9 | 49 |  |
| TTYH1 | p.E440fs | 0.130 | 6 | 31 |  |
| TTYH1 | p.440_441insH | 0.130 | 6 | 30 |  |
| KIF5C | p.K151fs | 0.200 | 3 | 16 |  |
| LY75-CD302 | p.T1393I | 0.188 | 3 | 18 |  |
| BARD1 | p.P24S | 0.087 | 4 | 59 |  |
| KRTAP10-12 | p.P92S | 0.133 | 5 | 38 |  |
| NFXL1 | p.P246L | 0.176 | 3 | 20 |  |
| PLA2G7 | p.R92H | 0.188 | 3 | 18 |  |
| NAT2 | p.R268K | 0.200 | 7 | 28 | p.R268K |
| AQP7 | p.Y64F | 0.068 | 7 | 88 |  |
| KIAA1984 | p.N421T | 0.125 | 4 | 42 |  |
